# Radiotherapy Both Promotes and Inhibits Myeloid-Derived Suppressor Cell Function: Novel Strategies for Preventing the Tumor-Protective Effects of Radiotherapy

**DOI:** 10.3389/fonc.2019.00215

**Published:** 2019-04-02

**Authors:** Suzanne Ostrand-Rosenberg, Lucas A. Horn, Nicholas G. Ciavattone

**Affiliations:** ^1^Department of Pathology and Huntsman Cancer Institute, University of Utah, Salt Lake City, UT, United States; ^2^Department of Biological Sciences, University of Maryland Baltimore County, Baltimore, MD, United States; ^3^Marlene and Stewart Greenebaum Comprehensive Cancer Center, University of Maryland, Baltimore, MD, United States

**Keywords:** radiotherapy-induced immune suppression, programmed death ligand 1 (PD-L1), myeloid-derived suppressor cells (MDSC), bi-specific T cell engager (BiTE), solubilized CD80

## Abstract

Cancer immunotherapies aimed at neutralizing the programmed death-1 (PD-1) immune suppressive pathway have yielded significant therapeutic efficacy in a subset of cancer patients. However, only a subset of patients responds to antibody therapy with either anti-PD-1 or anti-PD-L1 antibodies. These patients appear to have so-called “hot” tumors containing tumor-reactive T cells. Therefore, checkpoint blockade therapy may be effective in a larger percentage of cancer patients if combined with therapeutics that also activate tumor-reactive T cells. Radiotherapy (RT) is a prime candidate for combination therapy because it facilitates activation of both local antitumor immunity and antitumor immunity at non-radiated, distant sites (abscopal response). However, RT also promotes tumor cell expression of PD-L1 and facilitates the development of myeloid-derived suppressor cells (MDSC), a population of immune suppressive cells that also suppress through PD-L1. This article will review how RT induces MDSC, and then describe two novel therapeutics that are designed to simultaneously activate tumor-reactive T cells and neutralize PD-1-mediated immune suppression. One therapeutic, a CD3xPD-L1 bispecific T cell engager (BiTE), activates and targets cytotoxic T and NKT cells to kill PD-L1^+^ tumor cells, despite the presence of MDSC. The BiTE significantly extends the survival time of humanized NSG mice reconstituted with human PBMC and carrying established metastatic human melanoma tumors. The second therapeutic is a soluble form of the costimulatory molecule CD80 (sCD80). In addition to costimulating through CD28, sCD80 inhibits PD-1 suppression by binding to PD-L1 and sterically blocking PD-L1/PD-1 signaling. sCD80 increases tumor-infiltrating T cells and significantly extends survival time of mice carrying established, syngeneic tumors. sCD80 does not suppress T cell function via CTLA-4. These studies suggest that the CD3xPD-L1 BiTE and sCD80 may be efficacious therapeutics either as monotherapies or in combination with other therapies such as radiation therapy for the treatment of cancer.

## Introduction

Checkpoint inhibitors that inactivate the programmed death-1/programmed death ligand-1 (PD-1/PD-L1) pathway protect T cells from anergy and apoptosis and have significantly improved the survival of cancer patients with certain types of malignancies. As a result, antibodies to PD-1 and PD-L1 are now FDA-approved for the treatment of Hodgkin's disease, melanoma, merkel cell, non-small cell lung, head and neck, gastroesophageal, bladder, urothelial, renal cell, and hepatocellular cancers, and are being tested in numerous other types of cancer.

Cancers with high mutation rates and *de novo* tumor-infiltrating lymphocytes have response rates of 53–87%, while tumors with lower levels of mutations have response rates of approximately 20% [reviewed in (1)]. Tumor cell mutations render tumor cells immunogenic, resulting in the activation of T cells which traffic to the sites of tumor [tumor-infiltrating T cells (TIL)]. T cell activation and function are characterized by many factors including the expression of PD-1 and by the production of interferon gamma (IFNγ), which is also a potent inducer of PD-L1. Therefore, inherently immunogenic tumors are more likely to be candidates for PD-1/PD-L1 antibody therapy, particularly if the mutations are present in the cancer stem cells and also expressed in the progeny of the stem cells (2).

TIL are a key component for the efficacy of PD-1/PD-L1 therapy; however, not all tumors have a high rate of mutation and do not contain TIL. Therefore, alternative strategies for increasing TIL are being developed. Radiotherapy (RT) is a prime candidate because it facilitates activation of anti-tumor immunity at both locally radiated and distant non-radiated sites (abscopal response) (3, 4). However, RT also promotes tumor cell expression of the checkpoint blockade molecule PD-L1 (5, 6). Multiple studies in mice (6, 7) and patients (8–10) have demonstrated that checkpoint blockade inhibitors (CBI) such as antibodies to PD-1 and PD-L1 delay tumor progression and increase overall survival, thus confirming the suppressive role of PD-1/PD-L1 activity. As a result, there is extensive interest and enthusiasm for combining checkpoint blockade immunotherapy with RT (3, 4, 11–16). Preclinical studies in mice support the concept that the combination of radiotherapy with checkpoint blockade has increased therapeutic efficacy (17, 18), and the few clinical studies completed to date suggest the combination approach will benefit cancer patients (19–23).

However, RT also promotes myeloid-derived suppressor cells (MDSC) (24), another potent immune suppressive mechanism. MDSC use a variety of mechanisms to suppress antitumor immunity; however, they also can express PD-L1, and RT increases MDSC expression of PD-L1 (5, 25). Given that RT enhances immunogenicity but also enhances immune suppression through increased MDSC and PD-L1, this review will summarize how RT induces immune suppression in the context of MDSC and PD-L1 and will describe two novel strategies for neutralizing this RT-induced immune suppression. This information may provide the basis for new approaches for treating cancer in combination with RT.

## Radiotherapy Activates the Immune System but also Drives Immune Suppression

Radiotherapy (RT) has been a staple of cancer treatment for some cancers for over a century. Traditionally it was thought that RT controls tumor progression through the induction of DNA damage which results in tumor cell death (26). DNA damage also causes lymphopenia (27) and therefore was considered a deterrent to antitumor immunity. However, T cells contribute to the regression of tumors following radiation (28), and local radiation facilitates the development of tumor-reactive T cells that home to the tumor microenvironment (29). Not only does radiation affect the local radiation site, but it can also limit/prevent progression of distant metastases. This phenomenon is known as the abscopal effect and is mediated by the immune system (30). These studies suggest that RT systemically activates tumor-reactive T cells and makes RT a logical therapy to combine with inactivation of the PD-1/PD-L1 pathway to increase patient responses.

However, RT also inhibits antitumor immunity by facilitating the development of immune suppressive cells, such as T regulatory cells (Tregs) (31), tolerogenic and immune suppressive dendritic cells (DC) (32), tumor-associated macrophages (TAMS) (33), tumor-associated neutrophils (TANs) (34), and MDSC (24), via a series of soluble molecules such as TGFβ (35), adenosine (36), VEGFA (37), CSF1 (24), and CCL2 (38). It is beyond the scope of this article to discuss all of these mechanisms, so the below discussion focuses on MDSC, which are present in virtually all cancer patients and are universally considered a major obstacle to cancer immunotherapies. Descriptions of the effects of RT on other immune suppressive cells and factors have recently been comprehensively reviewed (3, 39–41).

## Myeloid-Derived Suppressor Cells (MDSC)

MDSC are a diverse mixture of cells of myeloid lineage at intermediate stages of differentiation. There are two broad categories of MDSC: monocytic (M-MDSC) and granulocytic or polymorphonuclear (PMN-MDSC). These categories are defined based on their presence in the circulation. In humans M-MDSC are phenotypically CD11b^+^CD14^+^HLA-DR^−/low^ and PMN-MDSC are CD11b^+^CD14^−^CD15^+^or CD66b^+^HLA-DR^−/low^. Human M-MDSC may also express low levels of CD15. All MDSC are negative for the lineage markers characterizing non-myeloid cells. As apparent from their names, M-MDSC are mononuclear and PMN-MDSC are polymorphonuclear. A third category of human MDSC has recently been defined. These “early-stage MDSC” (eMDSC) are CD33^+^HLA-DR^−^ and do not express either CD14 or CD15. Mouse M-MDSC are CD11b^+^Ly6C^+^Ly6G^−^ and PMN-MDSC are CD11b^+^Ly6G^+^Ly6C^−^. Since the mouse marker Gr1 can include both Ly6C and Ly6G, total mouse MDSC are sometimes phenotyped as CD11b^+^Gr1^+^ (42). PMN-MDSC and neutrophils share the same surface markers, so phenotype alone is not sufficient for identifying either human or mouse cells as MDSC. Human PMN-MDSC and neutrophils have different densities so that PMN-MDSC tend to band with mononuclear cells at lower densities in Ficoll gradients, while neutrophils pellet at a higher density (43). However, the definitive characteristic of both M-MDSC and PMN-MDSC is their ability to inhibit the activation and function of T cells (44, 45). Mouse MDSC have been functionally characterized in more detail than human MDSC. In the context of tumor immunity they have also been shown to (i) polarize macrophages toward an M2-like pro-tumor phenotype (46), (ii) inhibit naïve T cell trafficking into lymph nodes and thereby prevent priming (47, 48); (iii) prevent T cell expansion by sequestering cysteine (49); (iv) drive the accumulation of Tregs (50); and (v) inhibit natural killer cell function (51).

MDSC arise in the bone marrow (and spleen of mice) in response to a variety of pro-inflammatory signals produced by tumors and host cells within the tumor microenvironment (44). The dominant driving factors are proinflammatory mediators such as IL-1β (52, 53), IL-6 (54), TNFα (55), prostaglandin E2 (56), high mobility group box protein 1 (57), and indole-amine2,3 dioxygenase (58). The cells traffic through the circulation and are chemoattracted to the tumor microenvironment by a series of chemokines such as CCL2 and CXCL2 that are present in the tumor microenvironment. Once in the tumor, hypoxia increases the suppressive potency of MDSC which is predominantly driven by the transcription factor STAT3 (59). MDSC have a relatively short half-life and M-MDSC can differentiate into non-immune suppressive myeloid cells (45). However, there is strong homeostatic regulation such that MDSC are rapidly replenished (60). A comprehensive discussion of MDSC induction and function can be found in several recent excellent review articles (61–63).

## Impact of RT on MDSC

Since RT induces a local inflammatory response including molecules such as C5a (64) which is a classical inducer of MDSC (65), it is not surprising that RT may induce the accumulation of MDSC. Cervical cancer patients receiving conventional fractionated RT (CFRT) showed an increase in levels of circulating MDSC along with reduced antigen presenting cell activity (66). In a mouse study using several prostate cancer cell lines, fractionated low dose RT caused an increase in MDSC in the blood, spleen, and lymph nodes. The effect was mediated by DNA damage that caused the ABL1 kinase to translocate to the nucleus where it bound to the promoter region of the CSF1 gene. The resulting increase in circulating CSF1 increased myeloid cell levels. Confirming the mouse studies, CSF1 was also elevated in the circulation of prostate cancer patients treated with RT (24). Tumor radioresistance via the induction of MDSC has also been attributed to RT-mediated activation of the Stimulator of Interferon genes (STING) pathway. Local radiation of tumor-bearing mice resulted in tumor cell production of the type 1 interferon IFNβ which, in turn, induced CCL2, CCL7, and CCL12 and chemoattracted CCR2^+^ M-MDSC to the tumor microenvironment (67).

MDSC levels have also been suggested as potential prognostic indicators of disease outcome. Following CFRT, hepatocellular carcinoma patients with high levels of M-MDSC have a poor prognosis (68).

MDSC have also been reported to have radioprotective activity. MDSC produce high levels of arginase 1 (Arg1). Arg1 promotes tumor progression by degrading arginine, an essential amino acid for T cell activation and function (69). Arginine is also the substrate for the production of nitric oxide (NO) which is generated by NO synthase (iNOS or NOS_2_). Under hypoxic conditions within solid tumors NO is a radiosensitizer that acts by reducing mitochondrial respiration (70). In an *in vitro* co-culture/radiation system using mouse and human tumor cells, Arg1-producing MDSC displayed radioprotective activity by reducing arginine and NO (71).

RT can also reduce MDSC levels, an effect that appears to require high dose ablative RT rather than multiple lower dose treatments. In studies with mice, ablative hypofractionated RT (AHFRT), but not CFRT, reduced the levels of intratumoral hypoxia, MDSC, and VEGF, and reduced MDSC expression of PD-L1 and VEGF receptor. Since hypoxia is a driver of PD-L1 expression (72) and VEGF is an inducer and chemoattractant for MDSC (73), the authors concluded that AHFRT reduced MDSC levels and function by decreasing intratumoral hypoxia and VEGF (74). In another mouse study, therapy with a single dose of ablative RT combined with anti-PD-L1 antibody therapy activated CD8^+^ T cells that subsequently decreased MDSC levels. CD8 T cell-mediated killing was by the production of TNFα (5), which is surprising since TNFα is an established inducer of MDSC (55). Another mouse study using a single high dose radiation treatment similarly resulted in elimination of MDSC. In this system, the high dose irradiation generated CD40L^+^CD4^+^ T cells and CD8^+^ dendritic cells that through cross-priming activated CD8^+^ T cells producing IFNγ (75).

**Figure 1:**
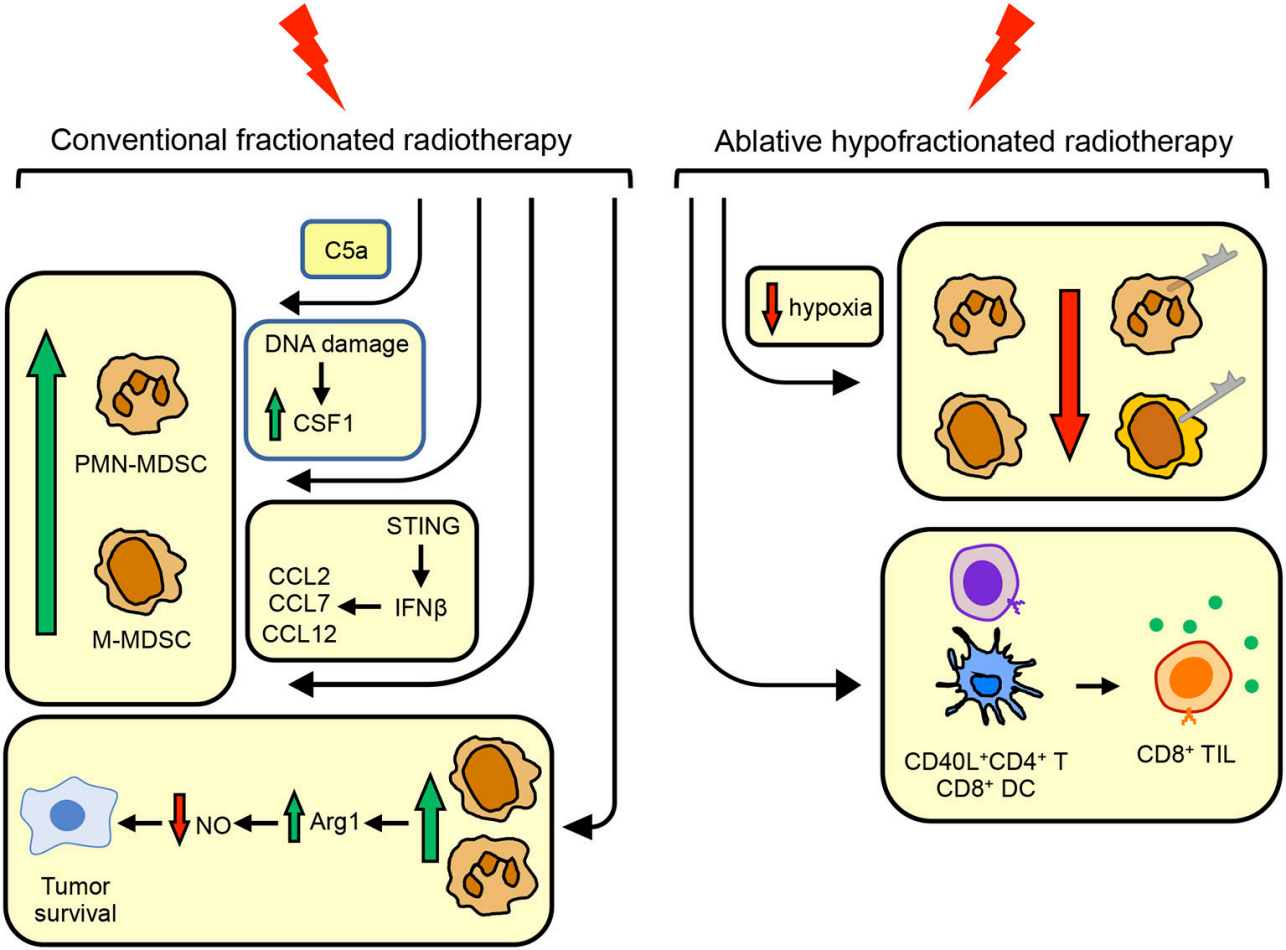
Conventional fractionated radiotherapy (CFRT) increases MDSC while ablative hypofractionated radiotherapy (ABHRT) decreases MDSC. CFRT increases the quantity of MDSC by (i) inducing the complement component C5a; (ii) causing DNA damage resulting in the up-regulation of CSF1; or (iii) signaling through STING to increase IFNβ which up-regulates CCL2, CCL7, and CCL12, chemoattractants for MDSC. MDSC up-regulated by CFRT facilitate tumor cell survival by their production of arginase 1 which decreases nitric oxide, a radiosensitizing molecule. ABHRT enhances antitumor immunity by reducing intratumoral hypoxia which decreases the quantity of MDSC and MDSC expression of PD-L1, resulting in increased levels of CD40L^+^CD4^+^ T cells and CD8^+^ DC which activate CD8^+^ TIL.

In conjunction with the findings of others for T cell responses and antitumor immunity (76), it appears that in contrast to CFRT, AHFRT may generate a more effective abscopal response and better antitumor immunity by limiting the accumulation of MDSC. If AHFRT is sufficient to eliminate MDSC, then additional strategies for reducing MDSC in patients receiving RT may not be needed. However, if AHFRT does not sufficiently eliminate MDSC or prevent MDSC up-regulation of PD-L1 (and potentially other ligands for checkpoint receptors), then additional therapies targeting these cells will be necessary. Figure 1 summarizes the conditions that drive the accumulation and function of MDSC, and the impact of CFRT and ABHRT on the generation of MDSC.

## A Bi-Specific T Cell Engager (BiTE) Activates T Cells That are Cytotoxic for PD-L1^+^ Tumor Cells

BiTEs are designed to activate T cells via CD3 and simultaneously bind to tumor cells via a tumor antigen. They are single chain recombinant proteins that contain the V_H_ and V_L_ regions of an anti-CD3 mAb attached by a short linker to the V_H_ and V_L_ regions of a mAb that reacts with a tumor antigen (77, 78). The first BiTE, Blinatumomab, specific for CD19, was FDA-approved for clinical use in 2014 (79). Our CD3xPDL1 BiTE uses the V_H_ and V_L_ regions of anti-CD3 mAb in combination with the V_H_ and V_L_ regions of the human anti-PD-L1 mAb 4A12 (80) to activate T cells and target them to PD-L1^+^ tumor cells. As with other BiTEs, the CD3xPDL1 BiTE has the potential to generate large numbers of cytotoxic CD3^+^ T cells regardless of T cell receptor expression or MHC genotype, and without costimulation, since the activation occurs via CD3 (81–83).

Binding studies using flow cytometry as the readout demonstrated that the ~55KDa CD3xPDL1 BiTE binds to CD3^+^ human peripheral blood mononuclear cells (PBMC), and to PD-L1^+^ human melanoma, chronic myelogenous leukemia, and lung adenocarcinoma cell lines, but not to CD3^−^ or PD-L1^−^ human tumor cells. Surface plasmon resonance studies indicated that the BiTE bound to CD3 with a dissociation constant of 2.4 × 10^−10^ and to PD-L1 with a dissociation constant of 1.28 × 10^−11^. The ability of the BiTE to simultaneously bind to CD3^+^ T cells and to PD-L1 was shown by detecting bound PD-L1-Fc to the BiTE-coated PBMC. When incubated with the BiTE in the presence of PD-L1^+^ tumor cells, PBMC from healthy human donors were activated as assessed by expression of the activation markers CD69 and CD25, their proliferation, and their production of IFNγ. Importantly, the BiTE-activated healthy donor PBMC were more cytotoxic for PD-L1^+^ tumor cells than PBMC activated by anti-CD3 mAb by itself, while PD-L1^−^ cells were not lysed. *In vitro* depletion studies demonstrated that the CD3xPDL1 BiTE not only activated cytotoxic CD4^+^ and CD8^+^ T cells, but also activated CD3^+^ NKT cells (84).

Since cancer patients frequently have MDSC that inhibit T cell activation and function, the CD3xPDL1 BiTE was also tested for its ability to activate cytotoxic cells from small cell (SC) and non-small cell lung cancer (NSCLC) patients. Approximately 24–60% of the PBMC from these patients consisted of M-MDSC (CD11b^+^HLA-DR^−^CD14^+^) plus PMN-MDSC (CD11b^+^HLA-DR^−^CD15^+^). Despite the high levels of MDSC, the BiTE activated CD3^+^ cells that specifically lysed PD-L1^+^, but not PD-L1^−^ human tumor cells (84). MDSC can express PD-L1 (85), so the ability to lyse tumor cells even in the presence of high levels of MDSC is likely due to BiTE-mediated cytotoxicity of the MDSC. T regulatory cells were not tested in this study. However, since RT induces Tregs (86) and induced Tregs may express PD-L1 (87), the CD3xPDL1 BiTE may also eliminate these cells.

The CD3xPDL1 BiTE was tested for *in vivo* efficacy using immune deficient NSG mice reconstituted with PBMC from healthy human donors (“humanized” mice). Humanized mice were inoculated with a spontaneously metastatic human melanoma and 7 days later the mice were given CD3xPDL1 BiTE for 4 consecutive days and a final dose of BiTE 2.5 weeks later. BiTE treated, but not control mice, had expanded numbers of human CD3^+^ cells in their spleens, minimal numbers of MDSC, and significantly extended survival times (84).

**Figure 2:**
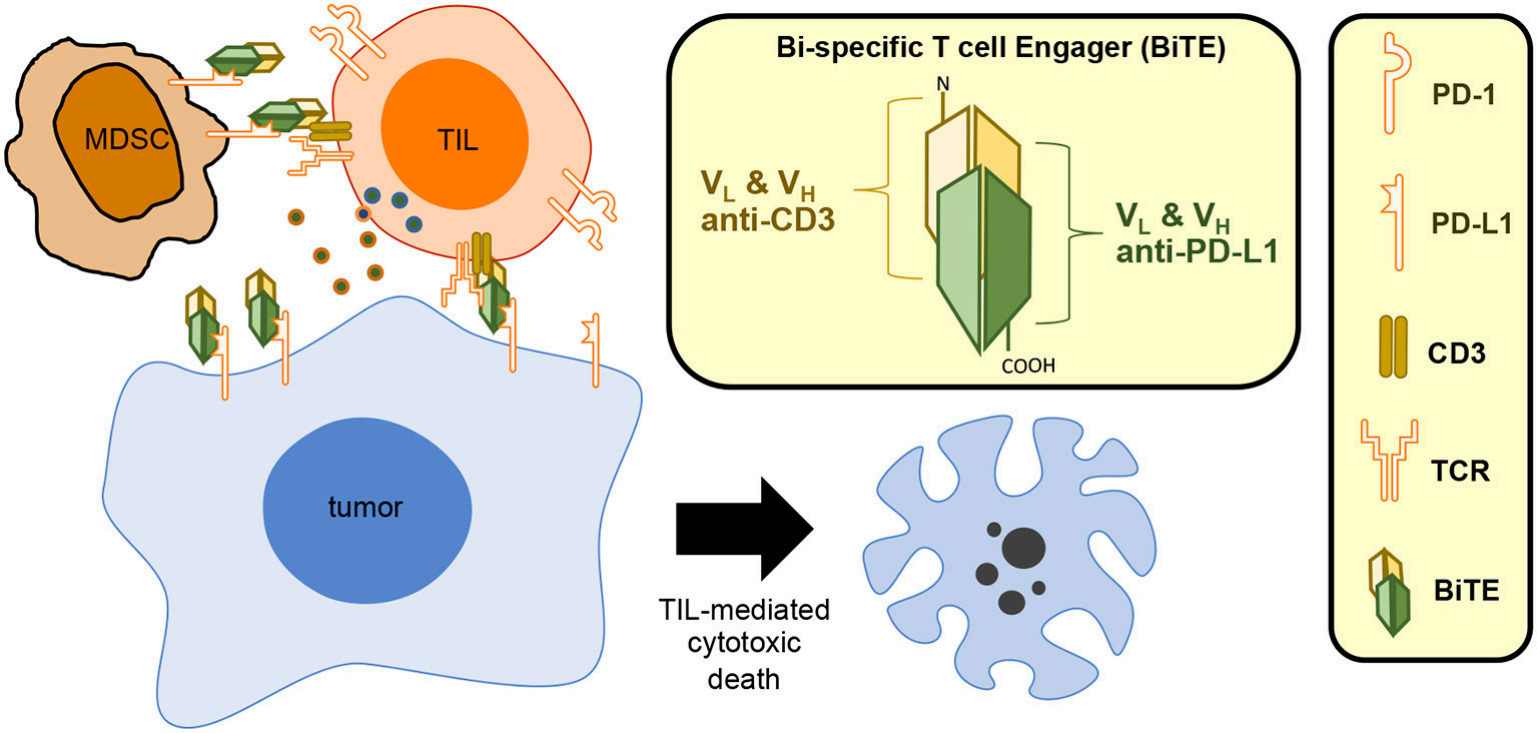
CD3xPDL1 BiTE blocks PD-L1 and induces T cell-mediated cytotoxic death. The CD3xPDL1 BiTE consists of the V_H_ and V_L_ regions of anti-CD3 and anti-PDL1 linked together to form a 55 kDa single chain structure. The CD3xPDL1 BiTE binds to PD-L1 on PD-L1^+^ tumor cells blocking interaction with PD-1 on T cells, thereby preventing PD-1 mediated T cell exhaustion. The BiTE simultaneously binds to CD3 on CD4^+^ T cells, CD8^+^ T cells, and NKT cells, activates the cells, and forms a cytotoxic synapse. The activated effector cells then kill the PD-L1^+^ tumor cells.

Collectively, these results suggest that the CD3xPDL1 BiTE might be a useful therapeutic to combine with other cancer immunotherapies and/or with RT. Since MDSC and PD-L1 can be induced by RT (5, 25, 66), and the BiTE expands TIL in response to PD-L1 while inhibiting MDSC, it would be interesting to determine if the CD3xPDL1 BiTE and RT synergize. Figure 2 shows graphically the structure and function of the CD3xPDL1 BiTE.

## The Soluble Form of CD80 (sCD80) Neutralizes PD-L1 Mediated Immune Suppression

PD-L1 not only binds to its receptor PD-1, but also binds to the costimulatory molecule CD80. Mutation analyses demonstrated that PD-1 and CD80 share overlapping binding sites on PD-L1, although the dissociation constant for PD-1/PD-L1 binding is approximately half that of the dissociation constant for CD80-PD-L1 binding (88, 89). This unexpected binding led to the hypothesis that CD80 might bind to PD-L1, thereby interfering with the binding of PD-L1 to PD-1 and facilitating and sustaining antitumor immunity (90). Initial studies of CD80-transfected human melanoma and lung adenocarcinoma cells that constitutively express PD-L1 or are induced by IFNγ to express PD-L1 suggested that CD80 inhibited the plasma membrane expression of PD-L1, despite the transfected cells containing PD-L1 mRNA and protein as assessed by RT-PCR and western blotting. However, the absence of detectable PD-L1 on the plasma membrane was subsequently shown to be due to CD80 sterically blocking the epitope on PD-L1 recognized by the anti-PD-L1 antibodies (91). The ability of CD80 to bind PD-L1 and prevent PD-1 binding was confirmed by assessing the binding of PD-1-Fc molecules to CD80^+^PD-L1^+^ and CD80^−^PD-L1^+^ human melanoma cells. CD80^+^PD-L1^+^ mouse tumor cells similarly did not bind PD-1-Fc, while CD80^−^PD-L1^+^ mouse tumor cells bound PD-1-Fc. Flow cytometry using an anti-PD-L1 antibody that recognized a non-CD80-dependent epitope revealed co-localization of PD-L1 and CD80 on the plasma membrane of human tumor cells. Whereas, CD80^−^PD-L1^+^ human tumor cells anergized activated PD-1^+^ human PBMC and inhibited their production of IFNγ, CD80^+^PD-L1^+^ human tumor cells prevented anergy and maintained IFNγ production (90). Mouse CD80^+^PD-L1^+^ tumor cells similarly maintained IFNγ production by activated PD-1^+^ mouse T cells (91). These results confirmed the hypothesis that CD80 might be a useful therapeutic for preventing the anergizing of any T cells via PD-1.

Since membrane-bound CD80 is not a feasible therapeutic, studies were initiated to determine if a soluble form of CD80 (sCD80 or CD80-Fc) had a similar function. Using four different human tumor cell lines, sCD80, but not an irrelevant Fc-linked protein, maintained IFNγ production by PD-1^+^ CD4^+^ and CD8^+^ T cells from human donors. A comparison of sCD80 to multiple anti-human-PD-L1 and anti-PD-1 antibodies demonstrated that sCD80 was more effective in maintaining IFNγ-producing activated T cells (91, 92). The latter finding in conjunction with CD80's known costimulatory activity, led to the hypothesis that sCD80 may be a dual agent that simultaneously blocks PD-1 suppression and costimulates through CD28. This hypothesis was confirmed by demonstrating that sCD80 maintained IFNγ production by PD-1^+^ activated CD28-deficient mouse T cells, but that the level of IFNγ was significantly higher for CD28^+/+^ PD-1^+^ T cells (92). sCD80 costimulation was further confirmed by western blotting and flow cytometry studies demonstrating that sCD80 activates EGR1-4 transcription factors in the CD28 activation pathway and phosphorylates MAPK, and NF-κB in the T cell receptor signaling pathway (93). Thus, sCD80 maintains T cell activation by simultaneously blocking PD-1 suppression and costimulating through CD28. Many tumor and other cells express PD-L1, so sCD80 has the potential to be a generally applicable reagent and is not limited to a specific type of tumor.

In addition to binding to PD-L1 and costimulating through CD28, CD80 also binds to the T cell-expressed co-inhibitory molecule CTLA-4, a receptor that decreases T cell activation and function. The mechanism of CTLA-4-mediated suppression is controversial. Although there is no known inhibitory motif in the cytoplasmic region of CTLA-4, it has been proposed that CTLA-4 functions by negative signaling into activated T cells. Alternatively, it has been suggested that CTLA-4 suppresses T cell function by acting as a “sink” or decoy receptor for CD80 and thereby scavenging CD80 and preventing it from binding to CD28 (94). To resolve if sCD80 suppressed through CTLA-4, CTLA-4^+^ activated human T cells were incubated with PD-L1^+^ human melanoma cells with or without sCD80 and/or blocking antibody to CTLA-4. Inclusion of anti-CTLA-4 antibody did not increase T cell activation, indicating that CTLA-4 suppression did not occur. Although T cell-expressed CTLA-4 did not impact T cell activation, inclusion of high levels of CTLA-4-Fc did reduce the ability of sCD80 to maintain IFNγ production, suggesting that mechanistically CTLA-4 serves as a decoy receptor (93).

sCD80 injected either intratumorally or systemically delayed tumor progression and extended survival time of syngeneic mice carrying the B16 melanoma or the CT26 renal cell carcinoma. Combination therapy of CT26-bearing mice with intratumoral sCD80 plus CpG further reduced tumor growth. Immunohistochemistry of tumors from systemically-treated mice with CT26 tumors revealed extensive TIL in the tumors of the sCD80-treated mice (93, 95). Studies with C57BL/6 CD28-deficient and PD-1-deficient mice carrying B16 tumors confirmed the earlier *in vitro* findings that sCD80 has the dual functions of inhibiting PD-1-mediated suppression while activating through CD28 (93).

**Figure 3:**
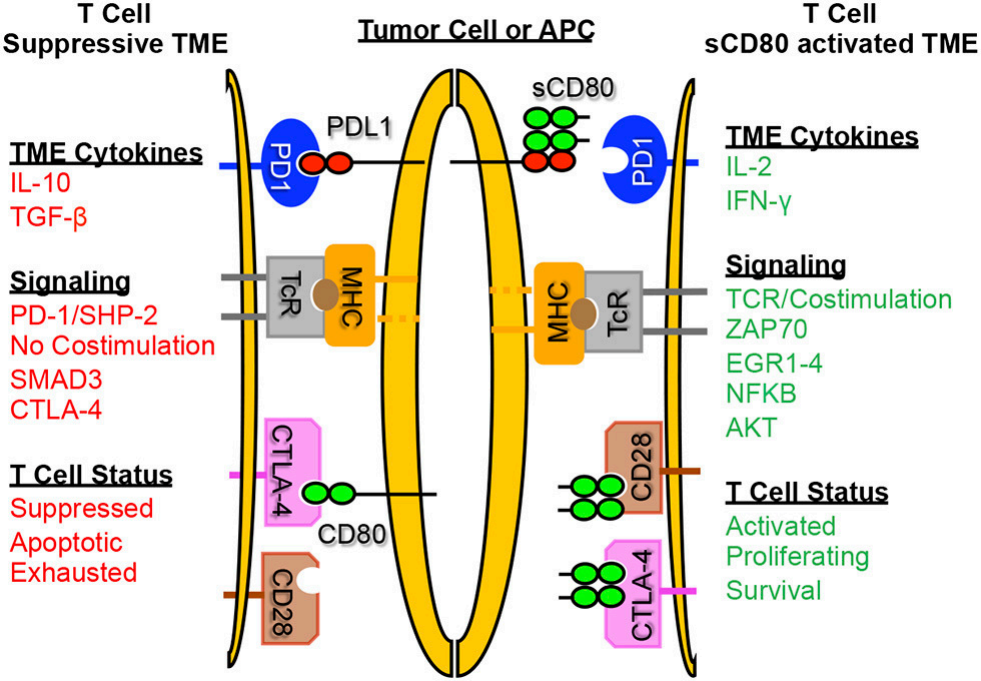
Soluble CD80 activates T cells, blocks PD-1 mediated immune suppression, and promotes anti-tumor immunity. sCD80 acts as a checkpoint inhibitor by blocking PD-L1 on tumor cells and antigen presenting cells while simultaneously binding and activating T cells through CD28. T cells activated by sCD80 have increased IFNγ and IL-2 production and upregulate TCR and CD28 signaling, resulting in an immune-reactive tumor microenvironment with T cell killing of target tumor cells.

Figure 3 is a graphic depiction of how sCD80 concurrently activates T cells via CD28 and prevents T cell anergy by inhibiting PD-L1/PD-1 binding.

## Conclusions

The use of antibodies to block the PD-1/PD-L1 pathway has been a major advance in the treatment of cancer patients. Since the efficacy of these antibodies depends on patients having tumor-reactive T cells that can be rescued and reactivated by the antibodies, it is essential to combine checkpoint blockade therapy with treatments that activate T cells in patients who do not have constitutively activated lymphocytes. Many cancer patients appear to be in this latter category since checkpoint blockade therapy is only effective in a subset of cancer patients. RT is a natural choice for improving the levels of activated T cells because it induces antitumor immunity both locally and systemically. However, RT can also drive PD-L1 expression and other immune suppressive mechanisms including MDSC. The CD3xPDL1 BiTE and soluble CD80 reagents described here not only inhibit PD-1/PD-L1 suppression, but also activate T cells. Therefore, if combined with RT, the CD3xPDL1 BiTE or sCD80 could synergize with RT to further drive T cell activation while concurrently neutralizing PD-1/PD-L1 immune suppression which may have been induced by the RT. New treatments could be developed where first, ablative hypofractionated RT is utilized to create an immunogenic tumor and reduce MDSC. Next, these novel therapies could be used to simultaneously block PD-L1, eliminate PD-L1^+^ tumor cells, and encourage expansion of TILs to eliminate the remaining tumor.

## Author Contributions

All authors listed have made a substantial, direct and intellectual contribution to the work, and approved it for publication

### Conflict of Interest Statement

The authors declare that the research was conducted in the absence of any commercial or financial relationships that could be construed as a potential conflict of interest.
